# Effects of Adding Intrathecal Epinephrine to Spinal Anesthesia on Anesthesia-Related Cesarean Delivery Outcomes: Should We Reconsider Its Use?

**DOI:** 10.7759/cureus.45147

**Published:** 2023-09-13

**Authors:** Milly T Rambhia, Alexander M DeLeon, Jeanette Bauchat, Eleanor Kenny, Candice Cuppini, Carmen Lopez, Alexander G Samworth, Jason Farrer, Robert J McCarthy

**Affiliations:** 1 Anesthesiology, Kaiser Permanente Mid-Atlantic Permanente Medical Group, Rockville, USA; 2 Anesthesiology, Northwestern University Feinberg School of Medicine, Chicago, USA; 3 Anesthesiology, Vanderbilt University Medical Center, Nashville, USA; 4 Anesthesiology, Rush University Medical Center, Chicago, USA

**Keywords:** hypotension, spinal anesthesia, length of stay, cesarean delivery, epinephrine

## Abstract

Background: Intrathecal epinephrine is used to increase the duration of spinal anesthesia for amenable surgical procedures anticipated to require additional time; however, in the ambulatory setting, it is associated with a prolonged time to post-anesthesia care unit (PACU) discharge. The current study's authors hypothesized that adding intrathecal epinephrine to spinal anesthesia for cesarean delivery would be associated with a dose-dependent prolonged post-anesthesia unit length of stay.

Methods: A single-center, retrospective study of patients undergoing repeat cesarean delivery under spinal anesthesia from 2011 to 2015 was conducted. Patients received spinal bupivacaine 12 mg, morphine 150 mcg, and fentanyl 15 mcg with no-epinephrine, 100 mcg, or 200 mcg of epinephrine. The primary outcome was recovery room length of stay. Secondary outcomes were surgical duration, intraoperative vasopressor use, perioperative opioids, and antiemetic use.

Results: Data were analyzed for 1,362 patients. Median recovery room stay was 123 min (interquartile range, 100 to 150) and was not different among groups. More women receiving epinephrine 200 mcg had ≥2 prior cesarean deliveries compared with no-epinephrine or 100 mcg. No significant differences in surgical duration or intraoperative opioids were identified among the groups. Median intraoperative vasopressor use was increased by 225 mcg of phenylephrine equivalents (99% CI, 25 mcg to 430 mcg) in the 100 mcg group and 250 mcg of phenylephrine equivalents in the 200 mcg group (99% CI, 75 mcg to 500 mcg) compared to no-epinephrine (*P*<0.001). Recovery room antiemetic and opioid analgesic administration were not different among groups.

Conclusions: Based on the results of this study, the addition of intrathecal epinephrine for women undergoing cesarean delivery increases intraoperative vasopressor use but does not prolong PACU length of stay, reduce intraoperative opioids, or increase antiemetic requirements postoperatively. The current study also demonstrated that surgical duration times were not different among the no-epinephrine, 100 mcg, and 200 mcg epinephrine groups.

## Introduction

Nearly one-third of all births in the United States and 19% worldwide are via cesarean delivery [1,2]. For women with a prior cesarean delivery, the repeat cesarean rate has been reported as greater than 90% [1,2]. Intrathecal epinephrine is an adjuvant to local anesthetics and opioids to potentiate analgesia via α2-adrenergic receptor stimulation, while its vasoconstrictive properties, primarily via α1-adrenergic receptor activation, increase block duration [3,4]. Two meta-analyses demonstrated the prolongation of sensory and motor blockade by adding intrathecal epinephrine to spinal anesthesia [5,6]. As a result, some practitioners add intrathecal epinephrine when performing anesthesia for higher-order (e.g., parity ≥2) elective cesarean deliveries.

The potential benefits of prolongation of a surgical anesthetic for higher-order cesarean deliveries must be weighed against the side effect profile, including a possible increased post-anesthesia care unit length of stay (PACU LOS). In the ambulatory surgical setting, intrathecal epinephrine has been shown to prolong the duration of sensory and motor blockade with a dose-dependent prolongation in time until discharge criteria are met [7,8]. Discharge from obstetric PACU typically requires evidence of spinal block resolution, such as two dermatome regression of sensory block or lower extremity movement. Increased PACU LOS due to epinephrine use could result in increased variable costs and reduced efficacy of resource utilization, personnel allocation, and lower patient satisfaction metrics [9]. Prior studies examining the addition of epinephrine to bupivacaine for spinal anesthesia for repeat cesarean delivery have primarily evaluated block onset, quality, and duration of the motor or sensory blockade but have not examined the effect on PACU LOS [8,10-12]. The authors of this report hypothesized that adding intrathecal epinephrine to bupivacaine, morphine, and fentanyl spinal anesthetics for cesarean delivery would prolong the PACU LOS in a dose-dependent manner.

## Materials and methods

The Institutional Review Board of Northwestern University approved this study. This manuscript was prepared using the Strengthening the Reporting of Observational Studies in Epidemiology (STROBE) guidelines [13]. The study design was a retrospective analysis of parturients presenting for repeat cesarean delivery under spinal anesthesia at Northwestern Prentice Women's Hospital from 2011 to 2015. The Northwestern Enterprise Database Warehouse (EDW) was used to capture data for this study.

The inclusion criteria were women presenting for repeat cesarean delivery who received spinal anesthesia. All included patients received hyperbaric spinal bupivacaine 12 mg (1.6 mL of 0.75% bupivacaine in 8.25% dextrose) with fentanyl 15 mcg, morphine 150 mcg with or without epinephrine (100 mcg or 200 mcg). Patients also received a phenylephrine infusion at 50 mcg/min adjusted to maintain the maternal blood pressure at baseline immediately after the intrathecal medications were administered per institutional routine. Excluded from data analysis were patients who developed post-partum hemorrhage (estimated blood loss > 1 L) or who were identified as higher risk for bleeding and thus prolonged surgical time (e.g., placenta previa, multiple gestation, prior post-partum hemorrhage). Exclusion criteria also included patients who underwent conversion to general anesthesia.

Data from the medical records included patient characteristics, intrathecal medications administered, perioperative vasopressor use, duration of surgery, intraoperative opioid use, PACU LOS, PACU opioids, PACU pain scores, and PACU antiemetic treatment. As Saravanan et al. [14] described, the relative potency ratio for phenylephrine to ephedrine 80:1 was used to calculate phenylephrine equivalents. Intravenous milligram morphine equivalents (MME) were calculated based on a ratio of 10:1 for fentanyl (mcg) and 6.67:1 for hydromorphone (mg).

The primary outcome, PACU LOS, was calculated from the surgical end-time to the time discharge criteria were met. The qualification for discharge was documented by an anesthesiologist's confirmation of two dermatomal regressions of the spinal anesthetic block, adequate pain control, and absence of nausea symptoms. The primary outcome was compared among groups using the Kruskal-Wallis H test and Dunn's multiple comparison test. Secondary outcomes, including duration of surgery, vasopressor use (phenylephrine equivalents), and perioperative opioid MME administration, were analyzed using the Kruskal-Wallis H test and Dunn's multiple comparison test. Ordinal and binomial outcomes, including intraoperative and PACU opioid and antiemetic use, were compared between groups using a Chi-squared test.

Effect size differences among groups for patient characteristics, intraoperative, and postoperative data were calculated using partial Omega squared for interval data and Cramer V for ordinal and nominal data. Confidence intervals for effect size estimates were determined using a 1000-sample bootstrap. All tests were 2-sided, and a P<0.01 was required to reject the null hypothesis. Data analysis was performed using R version 3.5.3 (The R Foundation for Statistical Computing, Vienna, Austria) and RStudio version 1.2.1335 (RStudio Team 2015, RStudio: Integrated Development for R. RStudio, Inc., Boston, MA).

The sample for this study was a convenience sample of cesarean deliveries at Prentice Women's Hospital, assuming an average yearly number of repeat cesarean deliveries of 300 and that 90% would meet the inclusion criteria. The estimated sample (n=450 per group) would achieve an all-pair power of 0.95 using the Kruskal-Wallis test with a target family-wise error rate of 0.01 to detect an increase of 5 minutes in the time to PACU discharge among the groups assuming the median discharge time for the no-epinephrine (no-EPI) group was 120 minutes. The group standard deviation was 30 minutes. Discharge time and standard deviation were determined from institutional records. Sample size calculations were made using PASS 15 Power Analysis and Sample Size Software (2017) (NCSS, LLC: Kaysville, Utah, USA, ncss.com/software/pass).

## Results

Data was analyzed for 1,370 women undergoing repeat cesarean delivery under spinal anesthesia. Fourteen cases were excluded due to erroneous charting of vasopressor use in the electronic record, and an additional 19 cases were excluded after further analysis revealed the patient had suffered extensive post-partum bleeding. The final data analysis included 1,337 patients, 400 patients in the no-EPI group, 524 in the 100 mcg epinephrine group, and 413 in the 200 mcg epinephrine group.

**Figure 1:**
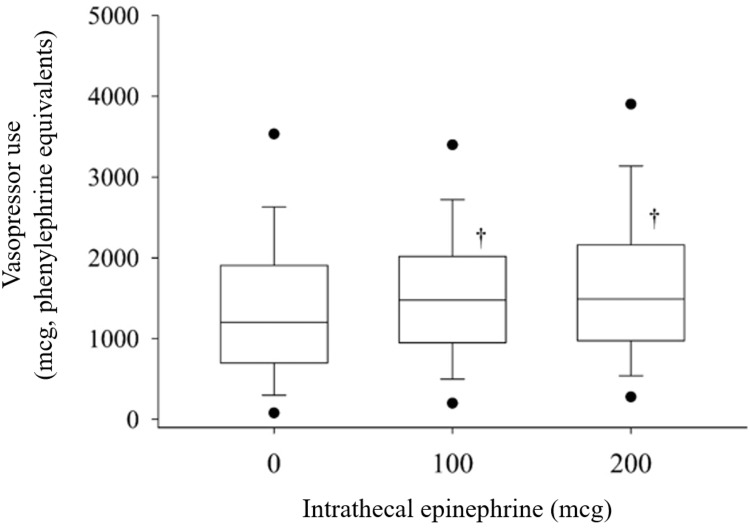
Box plots of intraoperative vasopressor use vs. intrathecal epinephrine dose. Box plots of intraoperative vasopressor use in phenylephrine equivalents by intrathecal epinephrine group. The horizontal bar represents median values, and the box depicts the interquartile (25th to 75th percentile) range. Whiskers represent the 10th and 90th percentiles and filled circles the 5th and 95 percentiles. † = different from no intrathecal epinephrine P<0.01. Vasopressor use was increased by 225 mcg (99% CI, 25 to 430, P<0.001) phenylephrine equivalents in the 100 mcg group and 250 mcg (99% CI, 75 to 500, P<0.001) phenylephrine equivalents in the 200 mcg group compared to the no-EPI group.

The study groups had no clinically significant differences in age, BMI, and gestational age (see Table 1). A greater percentage of women in the epinephrine 200 mcg group had parity ≥2 prior cesarean deliveries compared to the no-EPI or 100 mcg dose groups. The median surgical duration for the entire study group was 47 min with an interquartile range, IQR, of 37 to 59 min, which was 4 min (99% CI, 0 to 6, P=0.005) longer in the 200 mcg epinephrine group compared to the no-EPI group, and 3 min (99% CI, -0.5 to 5, P=0.015) longer in the epinephrine 200 mcg compared to the epinephrine 100 mcg group. There were negligible differences in the number of women that received or the median amount of intraoperative opioid analgesic administered among the groups. Intraoperative vasopressor use was increased by 225 mcg (99% CI, 25 to 430, P<0.001) of phenylephrine equivalents in the 100 mcg group and 250 mcg (99% CI, 75 to 500, P<0.001) of phenylephrine equivalents in the 200 mcg group compared to the no-EPI group (see Figure 1).

**Table 1:** Clinical characteristics of intrathecal epinephrine groups. IQR = interquartile range, *statistically significant

	Epinephrine 0 mcg (n=400)	Epinephrine 100 mcg (n=524)	Epinephrine 200 mcg (n=413)	P
Age, years (IQR)	34 (31 to 36)	34 (31 to 37)	35 (32 to 37)	0.24
BMI, kg/m^2 ^(IQR)	30.8 (27.6 to 34.7)	30.7 (27.5 to 34.9)	30.3 (27.5 to 34.9)	0.85
Gestational age, weeks (IQR)	39.0 (38.6 to 39.3)	39.0 (39.0 to 39.2)	39.0 (39.0 to 39.3)	0.70
Parity, N (%)				<0.001*
1	323 (81)	357 (67)	160 (38)	
2	54 (13)	140 (26)	252 (60)	
>2	23 (7)	27 (7)	11 (2)	

Post-anesthesia recovery room data are shown in Table 2. The median PACU LOS was 123 min (IQR, 100 to 150) overall. The difference in the median PACU LOS was -5 min (99% CI, -11 to 3, P=0.18) for the epinephrine 100 mcg epinephrine group and 1 min (99% CI, -8 to 10, P=0.77) for the 200 mcg epinephrine group compared with the no-EPI group, respectively. Only 9.1% of all patients received opioids in the PACU for pain control, with a negligible difference in effect size among the groups. The median dose of opioids received among all groups was 6.7 MME (IQR, 3.3 MME to 10.0 MME), with no statistically significant difference among groups. The difference in median numerical rating scale score (NRS) pain scores at PACU discharge were -1 (99% CI, of the difference -2 to 0, P=0.003) and -1 (99% CI, of the difference -2 to 1, P=0.02) for the epinephrine 200 mcg and the 100 mcg epinephrine groups compared to the no-EPI group, respectively. All patients received 30 mg intravenous ketorolac in PACU. Antiemetics were administered to 9.1% (99% CI, 7.0% to 11.1%) of the women in the PACU, and the rate was not statistically different among groups.

**Table 2:** Surgical and post-anesthesia care unit data. *statistically significant, IQR = interquartile range, PACU = post-anesthesia recovery room

	Epinephrine 0 mcg	Epinephrine 100 mcg	Epinephrine 200 mcg	P
PACU duration, min (IQR)	125 (100 to 155)	120 (99 to 145)	126 (103 to 154)	0.06
Duration of surgery, min (IQR)	45 (35 to 59)	46 (37 to 57)	49 (39 to 61)	0.006*
Number requiring intraoperative opioid (%)	105 (26)	131 (25)	137 (32)	0.05
Number requiring PACU opioid (%)	74 (18)	78 (15)	84 (19)	0.12
Number of patients with PACU nausea/vomiting requiring treatment (%)	36 (9)	44 (8)	44 (10)	0.59

## Discussion

The current study authors hypothesized that adding intrathecal epinephrine to bupivacaine, morphine, and fentanyl for spinal anesthesia would be associated with a longer PACU LOS. The results demonstrate no prolongation of recovery time in the setting of cesarean delivery. This finding contrasts with the non-obstetric related literature showing a prolonged motor blockade in patients who received intrathecal epinephrine [5,10,12].

In a meta-analysis of 24 clinical trials encompassing a variety of surgical procedures, de Oliveira et al. [5] reported a dose-dependent effect of intrathecal epinephrine on motor blockade. The analysis included 10 studies that examined the effect of 200 mcg doses of intrathecal epinephrine on the duration of the motor block and identified a weighted mean difference of 47.0 mins (99% CI, 34.0-59.9) in favor of epinephrine. These results are consistent with those found by Abouleish et al. [10], who reported a difference of 27 minutes in complete motor regression between the no-EPI group and the 200 mcg epinephrine group. Katz et al. [12] performed a randomized control trial in women undergoing repeat cesarean delivery of intrathecal epinephrine dose on the duration of spinal anesthesia using hyperbaric bupivacaine and morphine. The results showed a mean increase of 30 min (95% CI, 15 to 45 min) and 40 min (95% CI, 15 to 60 min) in T10 sensory block regression between the no-EPI and either 100 mcg or 200 mcg epinephrine groups, respectively. The 100 mcg epinephrine group had a median time of 150 min (95% CI, 135 to 150), while the no-EPI group had a median time of 120 min (95% CI, 105 to 120) [12].

Unlike discharge following ambulatory anesthesia, complete recovery of motor function is not necessarily an expectation post-cesarean delivery [1,9]. The time course and recovery characteristics in the obstetric PACU also differ from those of the post-surgical PACU. It is possible that other non-anesthetic factors, such as breastfeeding teaching and initiation, as well as neonatal care and evaluations, affect the time to discharge the mother from the PACU following cesarean delivery. Thus, the differences between the PACU course of a post-cesarean patient and an ambulatory surgery patient might explain the negative finding for differences in PACU LOS reported in the current study.

The rationale for adding epinephrine to a spinal anesthetic often includes the expectation that the surgical procedure requires additional time, as expected with higher-order repeat cesarean deliveries. Despite the presumed use of intrathecal epinephrine for repeat cesarean delivery being due to anticipated longer surgical duration, no clinically meaningful difference was identified in surgical time across groups presenting for a second or third cesarean delivery. More than 100 obstetricians perform cesarian deliveries at the institution of the current study, and thus, the surgical times are unlikely to be affected by a small sample of providers. In addition, operative times in the present study were comparable to those reported by Katz et al. [12]. Thus, the current study's findings did not support the perceived need for prolonging surgical anesthesia by adding intrathecal epinephrine.

Detrimental effects of epinephrine were observed in the current study and in the peer-reviewed literature, which included alterations in maternal hemodynamics requiring vasoactive medications. The findings of this study demonstrate a 20% increase in intraoperative vasopressor use in the groups receiving 100 or 200 mcg of intrathecal epinephrine compared to those with no-EPI. Hypotension was reported to be more likely with intrathecal epinephrine use than placebo (OR 1.55, 95% CI, 1.05 to 2.3) in a meta-analysis including 24 clinical trials and 1,271 subjects [5]. Another adverse effect of intrathecal epinephrine reported in the literature is increased nausea and vomiting. De Oliveira et al. reported an odds ratio of 2.41 (95% CI, 1.45-3.9) (number needed to harm = 10) of nausea and vomiting with the addition of intrathecal epinephrine based on 10 studies and 14 comparisons [5]. The mechanism of postoperative nausea and vomiting in this subset of patients may not be entirely attributable to the reduction of blood flow to emetogenic areas of the medulla in the setting of hypotension or to a pharmacokinetic delay in clearance of intrathecal opioids by epinephrine [1,2,15]. This suggests that the vasopressor infusion likely mitigated additional hypotension caused by intrathecal epinephrine in the intraoperative setting, which may explain the negative findings regarding nausea in the current study.

The results of this study should only be interpreted in the context of its limitations. This study was a retrospective analysis of clinical use based on provider preference at a single large quaternary care maternal hospital. While PACU LOS based on the timing of the attending anesthesiologist's discharge order is a timely and reliable measure of recovery from spinal anesthesia at the study's institution, these results may not be broadly generalizable as they may reflect specific institutional practice patterns. Additionally, although the investigators were able to quantify the amount of vasopressor required for each patient, the amount of pre-hydration, adherence to the use of prophylactic phenylephrine infusion, degree of hypotension as a percentage from baseline, or other institutional practice patterns may have affected the findings of this study. Nonetheless, given the large sample size and many providers, the authors believe the data accurately represents clinical outcomes following the use of epinephrine combined with spinal bupivacaine for repeat cesarean delivery.

## Conclusions

The current findings and a review of the literature suggest that although intrathecal epinephrine with hyperbaric bupivacaine dose does not significantly increase the PACU LOS, adverse effects of intrathecal epinephrine can be observed, including increased vasopressor use, hypotension, and increased incidence of postoperative nausea and vomiting. Additionally, the results of the current study did not support a clinically significant increase in surgery duration for patients who received intrathecal epinephrine. Thus, our findings suggest a limited rationale for adding epinephrine to spinal bupivacaine for repeat elective cesarean delivery, assuming similar surgical durations to those recorded in this report. Future studies should investigate factors that could warrant the addition of epinephrine to spinal bupivacaine for repeat cesarean delivery.
